# Switchgrass (*Panicum virgatum*) Intercropping within Managed Loblolly Pine (*Pinus taeda*) Does Not Affect Wild Bee Communities

**DOI:** 10.3390/insects7040062

**Published:** 2016-11-04

**Authors:** Joshua W. Campbell, Darren A. Miller, James A. Martin

**Affiliations:** 1 Department of Entomology and Nematology, University of Florida, 1881 Natural Area Dr., Gainesville, FL 32611, USA; 2 Weyerhaeuser Company, P.O. Box 2288, Columbus, MS 39704, USA; darren.miller@weyerhaeuser.com; 3 Warnell School of Forestry and Natural Resources, University of Georgia, 180 E Green Street, Athens, GA 30602-2152, USA; martinj@warnell.uga.edu

**Keywords:** loblolly pine, *Pinus taeda*, switchgrass, *Panicum virgatum*, pollinators, *Ceratina*, *Lasioglossum*, Mississippi, intensive forestry, forest management

## Abstract

Intensively-managed pine (*Pinus* spp.) have been shown to support diverse vertebrate communities, but their ability to support invertebrate communities, such as wild bees, has not been well-studied. Recently, researchers have examined intercropping switchgrass (*Panicum virgatum*), a native perennial, within intensively managed loblolly pine (*P. taeda*) plantations as a potential source for cellulosic biofuels. To better understand potential effects of intercropping on bee communities, we investigated visitation of bees within three replicates of four treatments of loblolly pine in Mississippi, U.S.A.: 3–4 year old pine plantations and 9–10 year old pine plantations with and without intercropped switchgrass. We used colored pan traps to capture bees during the growing seasons of 2013 and 2014. We captured 2507 bees comprised of 18 different genera during the two-year study, with *Lasioglossum* and *Ceratina* being the most common genera captured. Overall, bee abundances were dependent on plantation age and not presence of intercropping. Our data suggests that switchgrass does not negatively impact or promote bee communities within intensively-managed loblolly pine plantations.

## 1. Introduction

Over 87% of all angiosperms are dependent on pollination services (primarily derived from insects) [1] and approximately 75% of the main global food crops are dependent on insects for those services [2]. Numerous studies have suggested that pollinators are in decline for a variety of reasons including increased pesticide usage, agricultural practices, habitat fragmentation, invasive species, spread of pathogens, and climate change [3]. Many agricultural practices (e.g., plowing, planting monocultures, etc.) are considered harmful to pollinator communities [4,5]. However, pollinator communities have not been extensively studied in production forest systems. Furthermore, relationships between pollinators and novel forestry systems, such as intercropping or agro-forestry, are not understood.

Biofuel production has increased in recent years and, if current trends continue, more agricultural land will be converted to biofuel crops [6]. Corn (*Zea mays* L.) is the primary crop used for ethanol and biofuel feedstock, but other feedstocks may serve as surrogates to corn. The Energy Independence and Security Act of 2007 (U.S. Public Law 110–140) calls for 136 billion liters of ethanol to be produced by 2022, much of which is supposed to come from cellulosic sources [7,8]. Most of the current research centers around cellulosic plants, which include grasses and any other raw material composed of cellulose [6]. Several cellulosic grasses are being examined as potential replacements for corn, which includes switchgrass (*Panicum virgatum* L.), a perennial grass native to eastern North America, which has great biomass potential with limited ecosystem demand [9].

Along with various grasses, tree species such as loblolly pine (*Pinus taeda* L.) have been investigated as a potential cellulosic source of biomass. Although most loblolly pine is used for traditional forest products (e.g., pulp and lumber), it has been shown to be an economical source of ethanol [10]. Intensively managed pine plantations cover more than 32 million acres in the southeastern United States [11]. Intercropping switchgrass in the inter-bed area (space between rows of planted trees) of pine plantations may augment ethanol production while also limiting future land conversion to biofuel crops (e.g., corn).

In some cases, managed forests have been shown to be less biodiverse than natural forests but intensively managed forests do contribute to conservation services for many animals and plants [12]. However, managed forests are an important part of the landscape and how these forests are managed is integral to sustainability. Nonetheless, some taxa are more sensitive to forest management practices resulting in lower species richness [13]. Alternatively, species diversity of birds was only slightly lower in managed pine forests in New Zealand [14] and invertebrate communities were fairly similar in managed forests compared to natural woodlands in Great Britain [15]. Overall, scientific literature shows conflicting evidence how overall biodiversity changes between managed forests and natural forests [13,16,17]. Although switchgrass and pine are wind-pollinated crops and these species would not contribute greatly to diets of foraging pollinators, managed forests can provide nesting habitat and other structure needed by native bees and beneficial insects [18]. Soil disruption can harm ground nesting bees [4], but managed pine forests generally do not experience such soil disruption after site preparation. Managed pine forests can also supply dead stems or downed woody debris which also provide nesting structure for wood nesting bees [19]. Some bees are capable of constructing their own nests within various coarse woody debris, whereas other bee species depend on tunnels made by wood boring beetles or other insects for a suitable nesting site [20,21]. Landscapes that can augment native bees could provide pollination services for nearby crops and other plants that require insects for pollination. However, little is known about how biofuel feedstock intercropping could affect biodiversity [22] of bees in a managed forest system.

Although sustainability of intercropping has been examined, including effects on vertebrate [23,24,25] communities, little is known about how biofuel feedstock intercropping could affect biodiversity [22] of bees in a managed forest system. Therefore, we examined potential effects of intercropping switchgrass in intensively managed loblolly pine plantations on bee communities by testing whether stand age and intercropping influenced bee abundance and diversity. We hypothesized that intercropping switchgrass between loblolly pine rows would affect some bee species by reducing plant diversity in the interbed area. Thus, we predicted a decrease in bees in intercropped stands. However, we hypothesized that intercropping would mitigate reduction in herbaceous vegetation overtime caused by a closing pine canopy. Therefore, we predicted that bees would be positively affected by intercropping in older pine stands. We also hypothesized that our older pine stands may provide increased coarse woody debris that could be used as nesting sites for many wood/stem nesting bees.

## 2. Experimental Section

### 2.1. Site Location and Preparation

We conducted our study on an approximately 9600 ha landscape of intensively managed pine plantations, owned and managed by Weyerhaeuser Company. The younger pine stands (intercropped and non-intercropped; see below) we studied were study plots established and managed by Catchlight Energy LLC, a Chevron and Weyerhaeuser joint venture, in Kemper County, Mississippi, between late spring and summer during 2013 and 2014. The study area landscape was comprised of intensively managed loblolly pine plantations (70%), mature pine-hardwood stands (17%), mature hardwood stands (10%), and non-forested areas (3%) [26]. Tree height for young plantations was between 2 and 4 m compared to 8 and 10 m for the older pine plantations. Tree density for young and old pine plantations was approximately 1112 trees/ha.

We used three replicates of four treatments, each approximately 8 ha in size: 3–4 year old intensively managed pine plantations (hereafter, Young Pine Plantations), 3–4 year old intensively managed pine plantations with switchgrass grown between rows (hereafter, Young Intercropped Pine), 9–10 year old intensively managed pine plantations (hereafter, Older Pine Plantations) and 9–10 year old intensively managed pine plantations with switchgrass grown between rows (hereafter, Older Intercropped Pine). Pine seedlings were planted during winter 2010–2011 for the 3–4 year old plantations and in 2004 for the 9–10 year old plantations. Switchgrass was seeded between rows May–June in 2011 and 2012 in the 3–4 year old plantation whereas in the 9–10 year old plantations, it was seeded between rows in 2009 and annually harvested during fall or winter. Woody debris in all plots with intercropped switchgrass were displaced within the inter-beds and pushed to the sides using a v-blade plow (See [23] for the detailed description of all plantations and plots).

### 2.2. Insect Trapping

**Figure 1:**
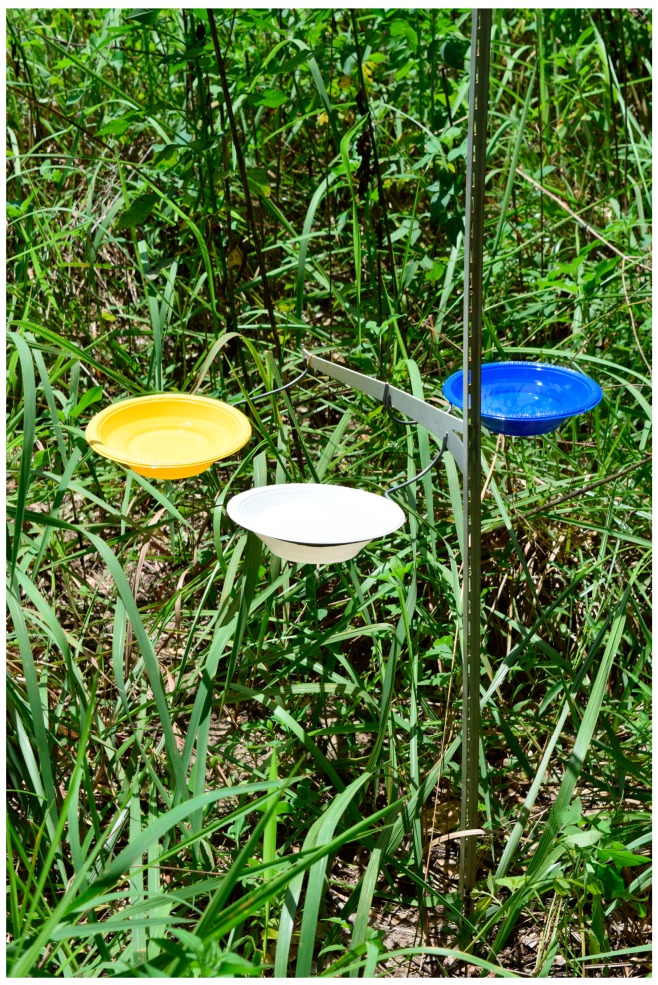
The pan trap “rack system” with the blue, yellow, and white bowls attached to brackets. Brackets were moved up as the grass grew during the growing season to consistently keep bowls at average vegetation height.

We captured insects using yellow, blue, and white pan traps (18 ounce Solo™ bowls) that contained soapy water. Although pan traps have potential limitations, they have been shown to be an efficient trapping method for pollinators in forested ecosystems of the southeastern United States [27,28]. Pan traps have also been used to collect bees within switchgrass monocultures and other wind-pollinated crops [29]. Within a plot, we established three sampling stations that were spaced at least >50 m from plantation edges to avoid edge effects and ≥50 m from the nearest station to minimize dependency among traps. Trap sets consisted of one of each bowl color placed on a “rack system” (Figure 1). The “rack system” consisted of an inverted shelf that allowed bowls, while in use, to be raised to average vegetation height throughout the growing season.

Beginning in May of 2013 and 2014, we collected pan trap samples twice a month (about 10–14 days apart) and concluded in late August of each year. During each trapping period, pan traps were active for 72 h intervals. We preserved collected insect samples in a 70% ethanol solution for future identification. We collected 15 samples during our two-year study (seven in 2013 and eight in 2014).

### 2.3. Statistical Analysis

We tested our hypotheses using GLM (Statistix 9 Program, Analytical Software, Tallahassee, FL, USA) to conduct one-way ANOVA’s with treatments as an independent variable and insect abundances as dependent variables. We averaged abundances across all sampling occasions (months) and subsamples (trap sites) for each plot. We conducted all abundance tests separately for each year given the dynamic nature of insect communities (*n* = 12 per year). We only tested bee genera/species in which a minimum of 50 specimens were collected for abundance analyses. We used a square root transformation to assure normality and homogeneity of variance. We used Tukey’s multiple range test to determine differences in relative abundances and diversity of bee genera among treatments. We used the Shannon-Wiener index (H’) to compare diversity of bee genera among treatments [30] and an ANOVA to compare Shannon-Weiner indices among plantation types. We examined diversity from a genus level because some bee genera (e.g., *Lasioglossum*) were difficult to identify to species. We used an alpha level of 0.05 for all tests.

## 3. Results

We captured 2507 bees from 18 genera (Table 1) during our two year study. The most common genera were *Lasioglossum* (51% of all bee captures), *Ceratina* (21%), *Augochlorella* (13%), and *Melissodes* (7%).

Most bees did not show a preference among treatments regardless of genera. However, bees in the genus *Lasioglossum* were more abundant within Young Pine Plantations compared to Older Pine Plantations in 2013 (F = 10.73, df = 3, 11, *p* = 0.003). Similarly, *Lasioglossum* was more abundant in Young Pine Plantations and Young Pine Intercropped compared to Older Pine Plantations and Older Pine Intercropped in 2014 (F = 16.49, df = 3, 11, *p* = 0.0009). *Augochlorella* bee abundances were similar among treatments in 2013, but in 2014, bees in this genus were more abundant in Older Pine Plantations compared to Young Pine Plantations (F = 3.92, df = 3, 11, *p* = 0.05). Numbers of bees captured within other genera were not significantly different (*p* > 0.05; Table 2). No differences (*p* > 0.05) in Shannon-Wiener indices of bee genera richness were observed among the treatments (Table 3).

## 4. Discussion

Our prediction of reduced bee abundance and diversity in young intercropped pine plantations was not well-supported. Also, our prediction of a positive impact of intercropping in older stands was not supported. Overall, bee communities were driven by forest age and not intercropping. In our study, most bees captured were ground nesting bees (e.g., *Lasioglossum*) which mass-provision pollen within underground tunnels for larvae. Most ground nesting bees are considered solitary but many will communally nest within suitable substrate [31]. Because the Young Pine Plantations and Young Intercropped Pine sites were in an early successional stage, they could have provided more bare ground allowing for suitable nesting structure. Forest management practices such as herbicide use and site preparation can temporarily increase amounts of exposed ground that can be used by ground nesting bees, possibly resulting in higher bee abundances [32]. Hardwood forests with cleared areas and open mature pine with a herbaceous understory were shown to have higher abundances and species richness of bees compared to dense young pine stands [28] possibly due to increased nesting sites and pollen/nectar sources. Overall, early successional forests have been shown to be important for native bees [33,34].

Intensively managed pine stands with and without intercropping of perennial grasses like switchgrass potentially allow for development of ground nesting conditions for bees because they require little to no cultivation after initial establishment causing minimal disturbance to the soil for ground nesting bees. We were not able to find any data exploring effects of soil disturbance on bees in forested systems. However, soil disturbance from plowing of agricultural land has been found to disrupt ground nesting bee nests [35]. For example, *Peponapis pruinosa*, an important pollinator of curcurbits, had a 3-fold density increase in no-tillage farms compared to tilled farms [36]. Hopwood [37] found that roadsides restored with native prairie vegetation had higher bee abundances and richness potentially due to reduced plowing which could have enhanced ground conditions for nesting bees. In large scale agricultural monocultures (e.g., corn), tilling also involves removing surrounding native vegetation, thus limiting plant and pollinator diversity [5,29]. In intensively managed forests, ground disturbance similar to plowing is limited to stand establishment. Therefore, it is reasonable to assume that any effects from soil disturbance in managed forests would be limited given a single disturbance event for a 27–35 year rotation length.

Wood and pith nesting bees (e.g., *Ceratina*, *Augochlora*) did not show differences among treatments. We hypothesized that the Older Pine Plantations and Older Intercropped Pine sites would support more wood/stem nesting bees due to the potentially higher abundance of coarse woody debris that could have been available for nesting structure. However, due to their small size, these bees could have also used small stems/twigs or decayed wood that may have been available within the Young Pine Plantations and Young Intercropped Pine treatments. Likewise, the larger bodied ground nesting bees (e.g., *Bombus*, *Melissodes*, etc.) showed no differences among treatments. Although foraging ranges for most bees are unknown [38], smaller solitary bees are known to generally have a small foraging range but large bodied bees have much larger foraging areas [39]. The likelihood of capture should be where they spend most of their time. Therefore, all sites provided equal nesting and foraging resources for these larger bodied genera resulting in no significant differences among the treatments. However, the pan trapping method does have biases and may not be the best trapping method for certain taxa [40] that could have been present.

We anticipated that the Older Pine Plantations and Older Intercropped Pine sites would have a more developed canopy and, hence, a more limited herbaceous understory whereas the Young Pine Plantations and Young Intercropped Pine sites would have allowed more sunlight penetration allowing for increased herbaceous plant diversity. Wheat [26] studied plant communities within the same plots we used and found that initially the Young Intercropped Pine contained lower plant diversity due to switchgrass out-competing other species and the use of glyphosate herbicide during establishment. However, by year two (when we started our study) plant diversity was not different among the young pine treatments [26]. Iglay et al. [41] looked at the older pine treatments and found that intercropping promoted a more diverse herbaceous plant community compared to non-intercropped plantations which contained more woody vegetation. Therefore, some significant trends in bee abundance could have been driven by differences in plant diversity within the plots.

Bee abundance and diversity was largely similar among treatments presumably because our study plots provided adequate nesting structure and foraging habitat to sustain populations. However, we captured high abundances of some genera (e.g., *Ceratina*) and very few individuals of other genera. Therefore, our experimental stands may only be providing adequate resources for a few of the common genera. Although no studies exist that examined bees in intensively managed loblolly pine plantations, bee abundance and species richness has been shown to be relatively lower in dense young pine plantations compared to other forest types in the southeastern United Sates [28]. Although we did not find increased richness of bees among any of the treatments, our data suggests that intercropping switchgrass within intensively managed loblolly pine plantations did not negatively affect overall bee diversity. Therefore, intercropping loblolly pine plantations with switchgrass may be one way to alleviate the need to convert more agricultural land into biomass crop production.

## 5. Conclusions

Establishing switchgrass or other grasses that could be used as a biofuel within the interbed area of pine plantations is an innovative approach. Native bees have been shown to be important pollinators of most flowering plants and crops. Researchers and land managers need to understand if novel forest management practices are disruptive to bee and other pollinator communities. Our results suggest that intercropping switchgrass within the interbed area of loblolly pine plantations in the southeastern United States does not appear to negatively or positively affect impact local bee diversity or abundances compared to traditional plantation management. Tree age and potentially tree canopy cover does, however, does cause differences in bee abundances. Using interbed areas of pine plantations may be a good way to maximize biofuel production while decreasing the need to convert other lands into biofuel crops; thus reducing widespread bee habitat loss.

## Figures and Tables

**Table 1:** Bee genera and species and total numbers captured in all traps within the four treatments in Kemper County, Mississippi, between late spring and summer during 2013–2014.

Family	Genus/Species	Total Captured
Andrenidae	*Perdita boltoniae* Roberstson	2
	*Andrena* spp.	5
Colletidae	*Hylaeus* spp.—including *H.mesillae* Cockerell, *modestus* Cockerell, *ornatus* Mitchell	22
Apidae	*Apis mellifera* L.	13
	*Bombus* spp.—including *B. Grisecolis* De Geer, *bimaculatus* Cresson, *pennsylvanicus* De Geer, *fraternus* Smith	65
	*Ceratina strenua* Smith	518
	*Melissodes* spp.—including *M. Bimaculata* Lepeletier, *comptoides* Robertson, *communis* Cresson, *agilis* Cresson	185
	*Melitoma taurea* Say	2
	*Svastra atripes* Say	2
	*Xylocopa virginica* L.	7
Halictidae	*Augochlora pura* Say	75
	*Augochlorella aurata* Smith	323
	*Halictus poeyi* Lepeletier*/lignatus* Say	6
	*Lasioglossum* spp.	1267
Megachilidae	*Coelioxys octodentata* Say*/sayi* Roberston	1
	*Hoplitis simplex* Cresson	7
	*Megachile* spp.—including *M. albitarsis* Cresson, *rotundata* Fabricius, *brevis* Say	6
	*Osmia georgica* Cresson	1

**Table 2:** Mean numbers (±SE) of the common genera of bees captured per plot, averaged across all subsamples (i.e., trap sites) and months, within the four treatments in Kemper County, Mississippi, between late spring and summer 2013–2014. Genera/family with an * indicate a significant difference at *p* ≤ 0.05. Within each genera, means followed by different letter(s) are significantly different.

Genera	Treatments			
	Young Pine Plantation	Young Pine Intercropped	Older Pine Plantation	Older Pine Intercropped
**2013**				
*Augochlora*	0	0.2 (0.05)	0.3 (0.07)	0.4 (0.08)
*Augochlorella*	0.3 (0.09)	0	0.5 (0.01)	0.6 (0.2)
*Bombus*	0.1 (0.04)	0.07 (0.03)	0.2 (0.06)	0.1 (0.04)
*Ceratina*	0.2 (0.06)	0.4 (0.08)	2.3 (0.6)	1.9 (0.6)
*Lasioglossum* *	3.6 (0.7) ^a^	2.3 (0.3) ^ab^	0.4 (0.09) ^b^	0.6 (0.1) ^ab^
*Melissodes*	0.2 (0.06)	0.4 (0.1)	0.4 (0.1)	0.3 (0.1)
**2014**				
*Augochlora*	0.1 (0.05)	0.02 (0.01)	0.1 (0.05)	0.02 (0.01)
*Augochlorella* *	0.4 (0.1) ^b^	0.6 (0.1) ^ab^	1.5 (0.3) ^a^	0.9 (0.2) ^ab^
*Bombus*	0.2 (0.06)	0.1 (0.04)	0.06 (0.03)	0.2 (0.05)
*Ceratina*	0.6 (0.1)	1.5 (0.4)	0.6 (0.2)	0.6 (0.1)
*Lasioglossum* *	5.9 (0.7) ^a^	4.6 (0.7) ^a^	0.5 (0.08) ^b^	0.9 (0.2) ^b^
*Melissodes*	0.5 (0.1)	0.3 (0.1)	0.4 (0.2)	0.2 (0.09)

**Table 3:** Mean Shannon-Wiener diversity indices (H’) and richness of genera (S) (±SE) from the four pine treatments. No significant differences in Shannon-Wiener diversity indices or richness were detected among the treatments at *p ≤* 0.05.

	Diversity Index	Young Pine Plantation	Young Pine Intercropped	Older Pine Plantation	Older Pine Intercropped
2013	S	7.3 (0.3)	7.7 (0.7)	7.3 (0.7)	7.3 (1.8)
	H’	0.562 (0.1)	0.676 (0.01)	0.743 (0.1)	0.772 (0.1)
2014	S	8.3 (1.2)	9.3 (1.2)	6.7 (0.7)	7 (1.0)
	H’	0.754 (0.1)	0.842 (0.04)	0.845 (0.1)	0.712 (0.05)
